# A Highly Sensitive Amperometric Glutamate Oxidase Microbiosensor Based on a Reduced Graphene Oxide/Prussian Blue Nanocube/Gold Nanoparticle Composite Film-Modified Pt Electrode

**DOI:** 10.3390/s20102924

**Published:** 2020-05-21

**Authors:** Jing Chen, Qiwen Yu, Wei Fu, Xing Chen, Quan Zhang, Shurong Dong, Hang Chen, Shaomin Zhang

**Affiliations:** 1 Key Laboratory of Biomedical Engineering of Ministry of Education, College of Biomedical Engineering and Instrument Science, Zhejiang University, Hangzhou 310027, China; keichen@zju.edu.cn (J.C.); 21715090@zju.edu.cn (Q.Y.); 21815055@zju.edu.cn (W.F.); cnxingchen@zju.edu.cn (X.C.); shaomin@zju.edu.cn (S.Z.); 2 Qiushi Academy for Advanced Studies, Zhejiang University, Hangzhou 310027, China; 3 Zhejiang Provincial Key Laboratory of Cardio-Cerebral Vascular Detection Technology and Medicinal Effectiveness Appraisal of China, Zhejiang University, Hangzhou 310027, China; 4 Neural Systems Group, Massachusetts General Hospital, Harvard Medical School, Charlestown, MA 02129, USA; qzhang@mgh.harvard.edu; 5 Zhejiang Laboratory, Hangzhou 310027, China; dongshurong@zju.edu.cn; 6 Key Laboratory of Micro-nano Electronic Devices and Smart Systems of Zhejiang Province, College of Information Science and Electronic Engineering, Zhejiang University, Hangzhou 310027, China; 7 MOE Frontier Science Center for Brain Research and Brain-Machine Integration, Zhejiang University, Hangzhou 310027, China

**Keywords:** microbiosensor, glutamate, glutamate oxidase, Prussian blue nanocubes, chitosan, amperometry

## Abstract

A simple method that relies only on an electrochemical workstation has been investigated to fabricate a highly sensitive glutamate microbiosensor for potential neuroscience applications. In this study, in order to develop the highly sensitive glutamate electrode, a 100 µm platinum wire was modified by the electrochemical deposition of gold nanoparticles, Prussian blue nanocubes, and reduced graphene oxide sheets, which increased the electroactive surface area; and the chitosan layer, which provided a suitable environment to bond the glutamate oxidase. The optimization of the fabrication procedure and analytical conditions is described. The modified electrode was characterized using field emission scanning electron microscopy, impedance spectroscopy, and cyclic voltammetry. The results exhibited its excellent sensitivity for glutamate detection (LOD = 41.33 nM), adequate linearity (50 nM–40 µM), ascendant reproducibility (RSD = 4.44%), and prolonged stability (more than 30 repetitive potential sweeps, two-week lifespan). Because of the important role of glutamate in neurotransmission and brain function, this small-dimension, high-sensitivity glutamate electrode is a promising tool in neuroscience research.

## 1. Introduction

L-glutamate is one of the excitatory neurotransmitters in the mammalian central nervous system (CNS), acting as a primary messenger molecule for approximately 90% of neurons [1,2]. Glutamatergic neurotransmission involves many normal brain functions, including memory, cognition, and learning processes. However, persistently elevated extracellular glutamate levels have been shown to be excitotoxic, eventually leading to the death of neurons. Meanwhile, the disruption of glutamate homeostasis has been confirmed to be linked to several neurological and mental disorders such as schizophrenia [3], pain syndromes [4], Parkinson’s disease [5], Alzheimer’s disease [6], epilepsy [7], depression [8], and anxiety disorder [9]. Reliable strategies are; thus, required in order to monitor the levels of glutamate and to illuminate the mechanisms of such diseases.

Many analytical techniques, such as fluorometric [10], spectrophotometric [11], chromatographic [12], and colorimetric methods [13], have been developed to measure glutamate in neurochemical analysis applications. Despite their high sensitivity, these methods still have not been widely applied in practical applications because of certain limitations, including high labor intensity, being overly time consuming, requiring expert handling, and involving sample pre-treatment [14]. Due to excellent analytical properties of resolution, sensitivity, and selectivity, electrochemical methods are considered as one of the most potential approaches for sensing of neurotransmitters [15]. At the same time, this method offers the advantages of simplicity and low expense. Electrochemical sensors can be easily tailored to estimate the concentration of glutamate and metabolites [16]. The electrochemical biosensors for glutamate have been mainly developed through two different enzymes—glutamate oxidase (GluOx) [17,18] and glutamate dehydrogenase (Gldh) [19,20]. GluOx involves flavina denine dinucleotide (FAD) catalyzed oxidation and the formation of hydrogen peroxide (H_2_O_2_) [21], and it is the most commonly used enzyme of glutamate for biosensors in applications of physiological environments. Many teams have been working on the glutamate electrode based on GluOx in recent years. However, both the size and the detection limit of these glutamate electrodes from previous studies still need more advancement for neurochemical applications [17,18,22,23]. The baseline concentration of glutamate in the extracellular space is relatively low (2–40 μM) [15,24]. Thus, in order to detect extremely low concentration changes in the extracellular fluid (ECF) of the brain, there is an urgent need to increase the sensitivity of the electrode to glutamate, while reducing the size of the electrode.

The availability of small-dimension commercial platinum (Pt) wires makes this material attractive for neuroscience applications. However, the high applied potential, often used to oxidize H_2_O_2_ with small-dimension platinum, will produce biofouling and interference by electroactive substances [25,26,27]. The electron-transfer mediator (ETM) is able to interact with the biological recognition component and to reduce aggregation by increasing electron transfer efficiency and reducing the oxidation potential. Within the ETM, Prussian blue (PB) is regarded as one of the most representative one to allow a rapid electron communication between the biomolecule and the electrode surface [28]. Nevertheless, the adhesion of PB requires high-quality active supports for immobilization [29,30,31]. Therefore, reduced graphene oxide (rGO) is introduced to increase the surface area of the electrode [32,33]. Meanwhile, rGO is endowed with ideal electrochemical properties, such as large a two-dimensional electrical conductivity and a large amount of edge-plan-like defects, enhancing the direct electron transfer between the enzyme’s active sites and the electrode [34]. Besides the choice of ETM, the immobilization and stability of enzymes are also issues that need to be considered for enzymatic biosensors [16]. Chitosan (CHIT) behaves as a substrate for enzyme immobilization while helping to maintain the activity of the enzyme, and it also offers the advantages such as good adhesion, high permeability, susceptibility, nontoxicity, and cheapness for chemical modification [31,35]. To further improve the electrical properties, gold nanoparticles (AuNPs) are becoming more and more widely used in the field of electrochemistry, as they can enhance electrode conductivity and facilitate electron transfer [36,37]. AuNPs, which can bond with the conjugated diene-based moieties of conducting polymer chains, also exhibit unique properties for speeding-up the polymerization of monomers [38,39]. Moreover, the composite materials of AuNPs and CHIT show the long lifetime usage of the biosensors [40]. 

In this context, electrochemical biosensors for detecting L-glutamate were designed by the simple electrochemical deposition of an rGO sheet, Prussian blue nanocubes (PBNCs), and AuNPs and CHIT onto a Pt wire. The developed electrode is physically characterized by field emission scanning electron microscopy (FE-SEM) and energy-dispersive X-ray spectrometer (EDS). The preparation and analytical parameters (pH and deposition cycles) are optimized. The stepwise surface analysis is characterized by cyclic voltammetry (CV) and electrochemical impedance spectroscopy (EIS) to analyze the electrochemical performance of the modified electrodes. The linear range, sensitivity, detection limit, specificity, and reproducibility are further investigated by chronoamperometry. To the best of our knowledge, this newly developed sensor is more promising for measuring glutamate in future neuroscience applications.

## 2. Materials and Methods

### 2.1. Reagents and Materials

L-glutamate oxidase (GluOx) from *Streptomyces* sp., L-glutamic acid (99%), Nafion^®^ perfluorinated resin solution (Nafion, 5 wt.%), gold (III) chloride trihydrate (HAuCl_4_⋅3H_2_O, 99.9%), ascorbic acid (AA, 99.7%), dopamine (DA, 98%), uric acid (UA, 99%), sodium sulfate anhydrous (Na_2_SO_4_, 99%), sulfuric acid (H_2_SO_4_, 99.99%), potassium chloride (KCl, 96%), phosphate buffer saline (PBS, pH = 7.2), potassium ferricyanide (K_3_[Fe(CN)_6_]), and potassium ferrocyanide (K_4_Fe(CN)_6_·3H_2_O) were all purchased from Sigma-Aldrich. Monolayer graphene oxide aqueous dispersion (GO, 10 mg/g) was obtained from C6G6 Technology Co., Ltd. (Hangzhou, China). Artificial cerebrospinal fluid (ACSF) was purchased from Beijing Leagene Biotechnology Co., Ltd. (Beijing, China). All solutions were stored at 4 °C and protected from light. Unless otherwise stated, all chemicals were used as received. 

### 2.2. Apparatus

All electrochemical experiments, including electrochemical impedance spectroscopy (EIS), cyclic voltammetry (CV), and chronoamperometry, were performed on a traditional three-electrode system with a CHI660E electrochemical workstation (Shanghai Chenhua Instrument Co, Ltd., Shanghai, China). The system consisted of a 1 mm commercial platinum wire counter electrode, an Ag/AgCl reference electrode, and the modified working electrode. The morphologies of the samples were characterized with a field-emission scanning electron microscope (FE-SEM; SU 8010, Hitachi, Tokyo, Japan), and the elemental composition of the electrode surface was analyzed by an energy-dispersive X-ray spectrometer (EDS; SU 8010, Hitachi, Tokyo, Japan). Fourier transform infrared spectrometry (FTIR) was carried out by the Fourier transform infra-red spectrophotometer (IRAffinity-1S, Shimadzu, Kyoto, Japan).

### 2.3. Fabrication of the Modified Electrode

**Figure 1:**
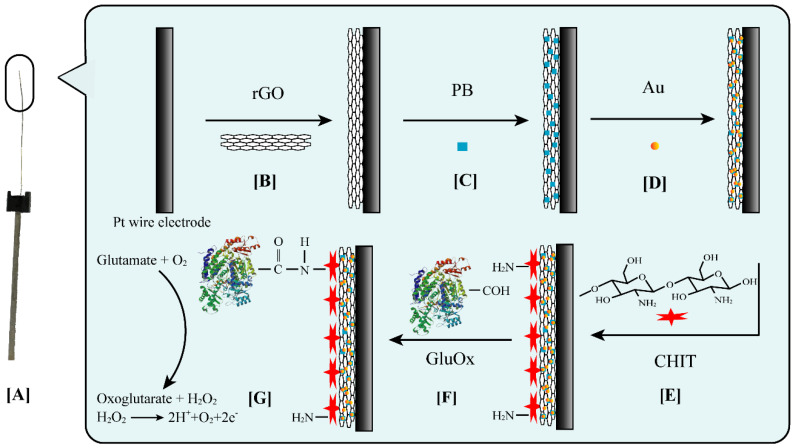
Schematic diagrams of the stepwise coating processes of the activated platinum wire. (**A**) The platinum wire fixed on a conductive connecting rod; (**B**) graphene oxide (GO) was reduced on the platinum surface; Prussian blue (**C**), gold (**D**), and chitosan (**E**) were electrodeposited; (**F**) immobilization of glutamate oxidase; (**G**) electrochemical redox of glutamate and hydrogen peroxide.

The fabrication of the sensor was based on a thin platinum wire with a diameter of 100 µm and a length of 6 mm, which was fixed on the base of the conductive connecting rod (Figure 1A). The following steps were mainly involved: cleaning, activation, and coating. First, the platinum wire was cleaned with ethanol, acetone, and deionized water, separately, for 0.5 h under ultrasonic agitation, in order to remove interfering substances such as grease from the platinum surface. After cleaning, the washed platinum wire was placed in 1 M H_2_SO_4_ and cyclically scanned from −0.5 to 1.3 V for 10 cycles, until the absorption and desorption peaks of both hydrogen and oxygen could be clearly observed, indicating the completion of the activation process. Figure 1 exhibits the stepwise coating processes of the activated platinum wire. Graphene oxide was then reduced on the surface of the activated platinum wire using the commercial single-layer graphene oxide aqueous dispersions, by cyclic scanning from −0.65 to 1.2 V (Figure 1B). The specific operation method and element characterization of the commercial graphene oxide dispersions have been introduced in detail in a previous work by our group [41]. Afterward, the electrode was performed by continuous potential cycling (12 cycles) between −0.15 and 1.3 V at a scan rate of 0.05 Vs^−1^ in a freshly prepared solution (20 mM FeCl_3_, 20 mM K_3_[Fe(CN)_6_], 0.1 M KCl, and 0.1 M HCl). During the scanning processes, redox reactions between Prussian blue with Prussian white, as well as Berlin green, occurred on the electrode surface, resulting in the deposition of Prussian blue particles (Figure 1C) [42]. Gold nanoparticles were then electrodeposited by constant potential scanning (six scans) in HAuCl_4_ (1 mM) and H_2_SO_4_ (0.5 M) at −0.2 V for 80 s (Figure 1D). After that, the electrode was dipped into a chitosan (0.5% in acetic acid) and KCl (1 M) mixed solution, while applying 20 successive deposition cycles at −0.15 to 0.20 V, at a scan rate of 20 mV/s (Figure 1E). The prepared electrode was placed into 100 μL glutamate oxidase (2.5 U/mL in 0.1 M PBS) and kept overnight at 4 °C for immobilization (Figure 1F). After the attachment of the glutamate oxidase, the electrode was finally dipped in a Nafion ethanol solution (0.5 wt.%) five times. The prepared electrode was washed with PBS (0.1 M) and stored at 4 °C before use. 

To investigate the individual effects of each modified material, rGO-Pt, AuNPs/PBNCs/rGO-Pt, and CHIT/AuNPs/PBNCs/rGO-Pt were prepared according to the partial procedure described above.

### 2.4. Analytical Procedure

Glutamate is oxidized by glutamate oxidase to generate hydrogen peroxide, and then hydrogen peroxide is oxidized at a specific potential, generating hydrogen ions and releasing electrons (Figure 1G). According to the following Randles–Sevcik equation [43], the properties of both the electrode and the analyte will affect the current generated by the oxidation reaction.

(1) $$I_{p\;}=2.69{\;\times \;10}^{5}{\;n}^{3/2}AD^{1/2}v^{1/2}C,$$

where *n* is the number of transferred electrons for the electrochemical system, *A* is the active surface area of the work electrode, *D* is the standard diffusion coefficient at 25 °C, *v* is the scan rate of cyclic voltammetry, and *C* is the concentration of the analyte. The equation provides a way to quantify the concentration of glutamate. At the same time, when the concentration of the solution is known, the effective surface area of the modified electrode can be calculated from the peak current (*I*_p_) measured by cyclic voltammetry.

The normalized peak current change (NPCC) was calculated by oxidation peak current intensities, using the following formula:

(2) $$\mathrm{NPCC}=(I_{a}-I_{b})/I_{b},$$

where $I_{a}$ and $I_{b}$ represent the characteristic peak currents of a 0.1 M PBS solution before and after the addition of analytes, respectively.

The impedance of the system was analyzed by fitting the Nyquist plot with the equivalent circuit through Zview (Solartron Analytical, Hampshire, U.K.), and the linear relationship in the experimental data was fitted by the linear model of GraphPad Prism^®^ Version 8 (La Jolla, CA, USA).

## 3. Results and Discussion

Figure 1 illustrates the design scheme and working principle of the modified sensor. For measuring the concentration of glutamate, glutamate oxidase was immobilized on the surface of the electrode, oxidizing glutamate to oxoglutarate and generating hydrogen peroxide. Hydrogen peroxide has a more significant electrochemical activity than glutamate. For fabricating the glutamate sensor, graphene oxide was first reduced on the surface of the platinum wire electrode, providing more sites for the subsequent attachment of nanoparticles. Prussian blue and gold nanoparticles were electrodeposited on the electrode surface in succession, which facilitated the oxidation of hydrogen peroxide. Subsequently, chitosan was electrodeposited on the electrode surface. The method of electrodeposition was simple, and the size of the particles formed is controllable. The side amine group of chitosan was covalently immobilized with the carboxyl group on the surface of the glutamate oxidase to form an amide bond (-CO–NH), which was conducive to maintaining the activity of glutamate oxidase and fixing the enzyme to the electrode more firmly [44]. Compared with the previously reported strategies, the sensors prepared by this method showed a superior performance in some aspects.

### 3.1. Morphology and Characterization

**Figure 2:**
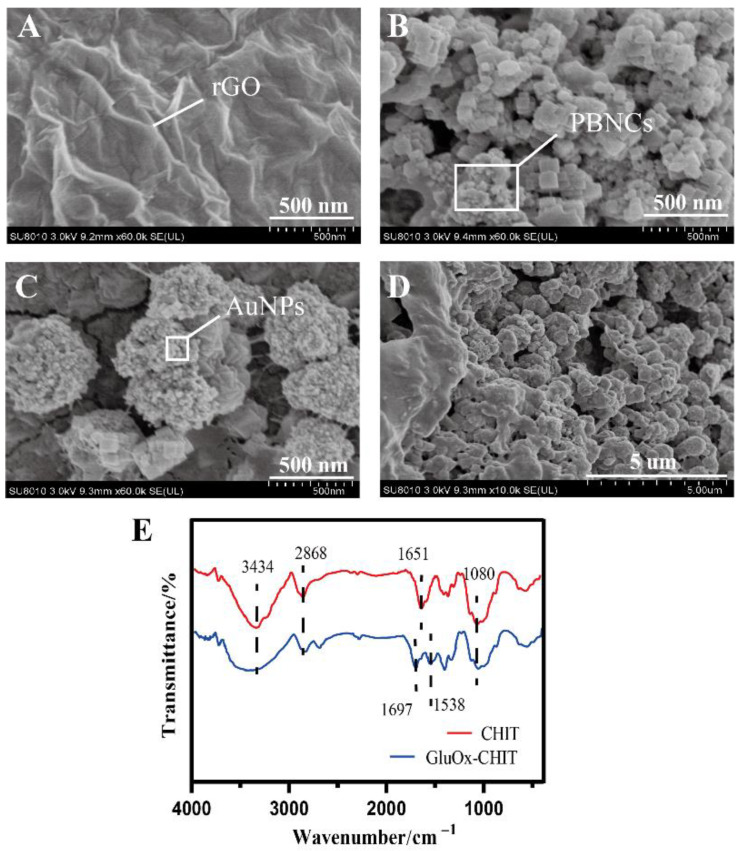
FE-SEM images at different phases of the modified processes of the electrodes. (**A**) rGO-Pt, (**B**) PBNCs/rGO-Pt, (**C**) AuNPs/PBNCs/rGO-Pt, and (**D**) modified electrode. (**E**) FTIR spectra of CHIT-Pt electrode (red curve) and GluOx-CHIT electrode (blue curve).

Figure 2A–D showed the surface morphological characters of the platinum wire electrode during the stepwise modification process by field emission scanning electron microscopy diagrams. The wrinkled structure on the GO-Pt electrode in Figure 2A indicated the presence of rGO. The carbon–oxygen ratio of rGO-Pt (about 11) was much larger than that of GO-Pt (about 1.5) in the EDS analysis, which confirmed that the oxygen-containing functional groups on GO were largely reduced. Compared with the original smooth surface of electrodes, the wrinkles of rGO increased the surface area, providing more active sites for the subsequent attachment of nanoparticles. In Figure 2B, the stacked nanocubes confirmed that Prussian blue was successfully modified on the electrode surface. With the electrodeposition of gold, the surface of Prussian blue cubes (~100 nM) was covered with smaller-sized gold nanoparticles (<50 nM). These nanoparticles offered enough free space and a large surface area, which supplied more electronic tunnels to facilitate electron exchange (Figure 2C). The final surface morphology of the modified electrode was shown in Figure 2D. The surface was tightly covered with nanoparticles, thereby increasing the number of immobilized enzymes within a specific surface area. A Nafion polymer membrane wrapped the enzyme in the outermost layer of the electrode, blocking the influence of interfering substances. 

The evidence of the attachment of glutamate oxidase arose from the FTIR results of CHIT modified electrode and GluOx-CHIT modified electrode. In Figure 2E, the four characteristic absorption bands of chitosan appeared at 3434 cm^−1^ for O–H and N–H stretching vibrations, 2868 cm^−1^ for C–H stretching vibrations, 1651 cm^−1^ for N–H bending vibrations, and 1080 cm^−1^ for C–O–C stretching vibrations [45]. When GluOx was introduced, new absorption bands appeared at 1697 cm^−1^ (C=O stretching of amide) and 1538 cm^−1^ (N–H bending of amide), indicating the formation of an amide bond (-CO–NH) and the successful immobilization of GluOx [46].

### 3.2. Optimization of Experimental Parameters 

Previous studies have showed that by adjusting the thickness of the surface coating, the sensitivity could theoretically be increased by an order of magnitude or more from the basic case [47]. We further investigated the following experimental parameters, including the buffer pH, the electrodeposition number of Prussian blue as well as gold, and the dipping number of Nafion, which affect the analytical performance in order to create the optimum conditions. 

**Figure 3:**
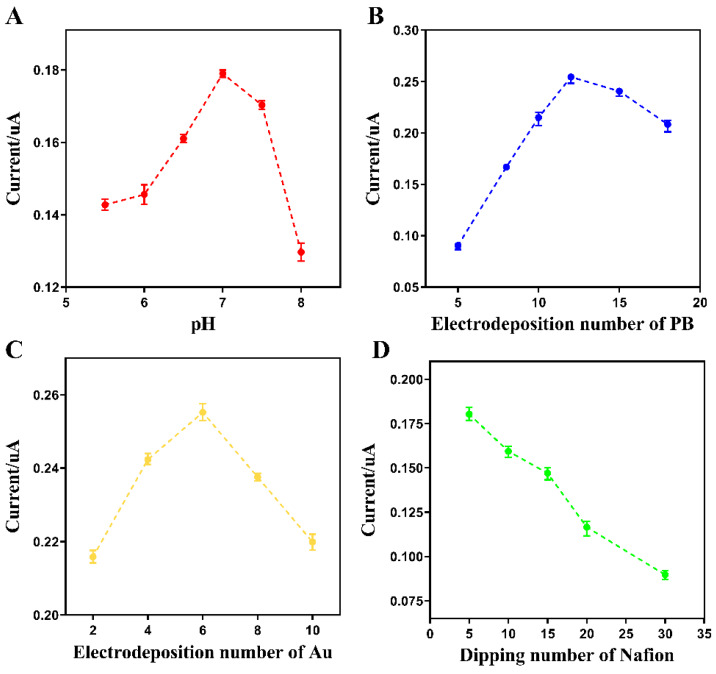
Variable optimization on the experimental conditions for the detection of glutamate (mean ± standard deviation (SD), *n* = 3). The corresponding response of the modified electrode through the relevant optimization of (**A**) the buffer pH, (**B**) the electrodeposition number of Prussian blue, (**C**) the electrodeposition number of gold, and (**D**) the dipping number in the Nafion solution.

From Figure 3A, the pH of the buffer varied from 5.5 to 8.0 when using the 0.1 M phosphate buffer solution. The current responses increased with the increasing pH, while the maximum current was found at pH 7. Thus, the phosphate buffer at pH 7 was selected for the experiment. 

The number of electrodepositions of Prussian blue and gold, as well as the number of dipping times of the Nafion solution, were used to refer to the thickness of the respective surface films. Observations indicate that the thickness of these films had an effect on the current response of the electrode. As the number of electrodepositions of Prussian blue increased, the current increased significantly. The current began to gradually decline after the twelfth time (Figure 3B). It was found that a low Prussian blue deposit led to a low coverage and thus minor improvement, while a high dosage of Prussian blue deposit led to a higher aggregation and lower electron transfer efficiency. The effect of gold deposition times on the performance of the electrode was similar to that of Prussian blue. Six depositions resulted in the most significant improvement in the electrode property (Figure 3C). In Figure 3D, the selective transmission membrane significantly reduced the amount of current detected by the electrode, as a result of the decrease of the analyte mass transfer rate within the Nafion film. The thickness of the surface selective transmission film should be minimized, while ensuring the selectivity of the electrode. Therefore, 12 electrodepositions of Prussian blue, six electrodepositions of gold, and five dipping processes of Nafion were used in this experiment.

### 3.3. Electrochemical Activities 

**Figure 4:**
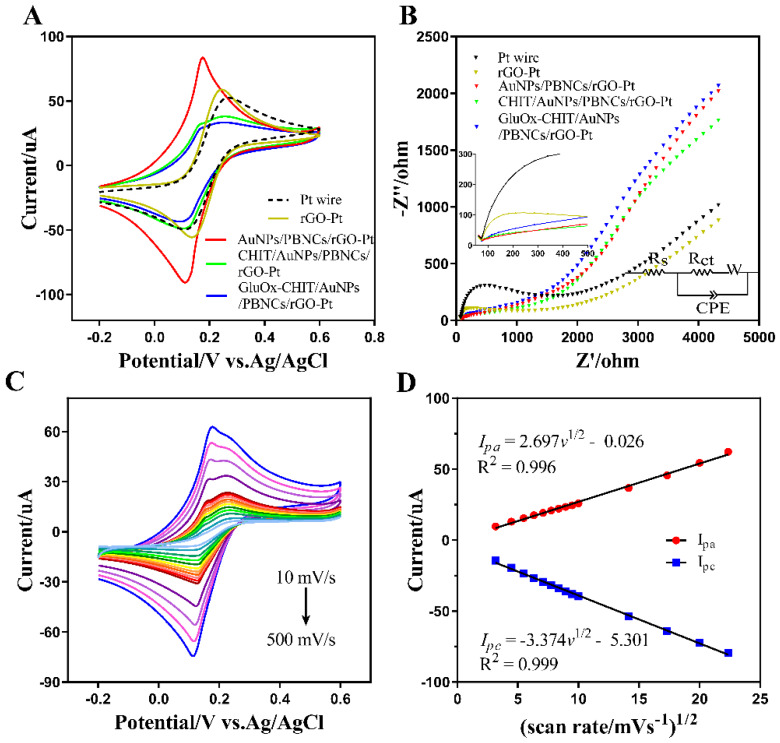
Electrochemical activities of the modified electrode. (**A**) Cyclic voltammetry (CV) responses and (**B**) Nyquist plots of Pt wire, rGO-Pt, AuNPs/PBNCs/rGO-Pt, CHIT/AuNPs/PBNCs/rGO-Pt, and GluOx-CHIT/AuNPs/PBNCs/rGO-Pt in the K_4_[Fe(CN)_6_]/K_3_[Fe(CN)_6_] system; (**C**) CV responses of the modified electrode for different scan rates from 10–500 mV/s; (**D**) linear plot for characteristic peak current intensities versus the square roots of the scan rates.

In order to investigate the effect of each modified material on the electrochemical activities of the electrode, the cycle voltammetry curves and electrochemical impedance spectra at different phases (rGO, AuNPs/PBNCs/rGO, CHIT/AuNPs/PBNCs/rGO, and GluOx-CHIT/AuNPs/PBNCs/rGO) of the modified processes of the electrode were recorded in a 10 mM K_4_[Fe(CN)_6_]/K_3_[Fe(CN)_6_] standardization system. The frequency range was 1–100 kHz, while the perturbation amplitude was 5.0 mV in the EIS analysis. A DC offset equal to the open circuit potential was applied to the system, keeping the system in a steady state. From Figure 4A, the rGO-Pt electrode showed more appreciable reduction and oxidation peaks than that of the bare Pt electrode, which was related to the superior electron transfer characteristics of rGO to promote the redox process of [Fe(CN)_6_]^3−/4−^. The AuNPs/PBNCs/rGO-Pt offered a higher redox peak current on the CV curve, indicating that this electrode provided a high electrocatalytic activity toward the [Fe(CN)_6_]^3−/4−^ redox couple. The potential difference between the anode and cathode peaks of it also gradually decreased, showing that the [Fe(CN)_6_]^3−/4−^ redox process on the electrode surface was more reversible after the modification of conductive nanocomposites. When the AuNPs/PBNCs/rGO-Pt was coated with chitosan and GluOx, the response current in the CV became smaller, which meant that chitosan and GluOx may have formed a hydrophobic protein layer that prevented the modified electrode from catalyzing the [Fe(CN)_6_]^3−/4−^ redox couple.

The Nyquist spectrum plot in Figure 4B exhibited a semicircle portion at high frequencies, signifying the electron and charge transfer resistance (R_ct_), and an inclined linear portion at low frequencies, corresponding to the diffusion process. The pattern of EIS could be fitted by the equivalent circuit shown in Figure 4B (bottom-right inset) with the resistance of the electrolyte solution (R_s_), the electron and charge transfer resistance (R_ct_), the Warburg element (W), and a constant phase element (CPE1) [24,48]. R_ct_ was affected by the dielectric and insulating feature at the interface between the electrode and electrolyte. The R_ct_ of the rGO-Pt wire (344 Ω) was found to be lower than that of the electrode before modification (825 Ω), showing that the electron transfer processes on the surface of the electrode became faster. More electron tunnels were served with further attachment of conductive nanomaterials (R_ct_ = 72.6 Ω), making it difficult to distinguish the semicircle, indicating a very fast electron transfer rate. The R_ct_ were calculated as 103.8 and 89.2 Ω as the modification of CHIT and GluOx, respectively. The increase in resistance was attributed to the fact that most biological macromolecules were poor conductors at low frequencies, hindering the transfer of electron [44]. These stepwise CV and EIS results were beneficial for analyzing the effect of various modified layers, and further confirmed that the materials were indeed attached to the surface of the electrode. The modified electrode in this paper exhibited a smaller charge transfer resistance than most glutamate microelectrodes in the previous work, attributing to the introduction of electron-transfer mediator and conducting metal nanoparticles which significantly improved the electron transmission speed on the surface of electrode [24,44,49,50,51].

To further calculate the effective surface area of the modified electrodes, the CV curves of the modified electrode were measured in 10 mM K_4_[Fe(CN)_6_]/K_3_[Fe(CN)_6_] at different scan rates (Figure 4C). In Figure 4D, the peak current intensities on the CV curves linearly increased with the rise of scan rates in the range of 10–500 mV/s. The linear equations fitted for the anodic (*I_pa_*) and cathodic (*I_pc_*) peak currents were expressed as $I_{pa}\left(\mu A\right)=2.697{\;v}^{1/2}\left(\mathrm{mV}/s\right)\;-\;0.026$ (R^2^ = 0.996) and $I_{pc}\left(\mu A\right)=-3.374{\;v}^{1/2}\left(\mathrm{mV}/s\right)\;-\;5.301$ (R^2^ = 0.999). According to the Randles–Sevcik equation (Formula (1)), the effective surface areas of the electrode could be estimated, while the mass transport was assumed to be only affected by the diffusion process. When the parameter *n* = 1, *v* = 0.1 V/s, and *D* = $6.7{\;\times \;10}^{-6}$ cm^2^/s, the surface areas of modified electrode was calculated to be 1.22 mm^2^, larger than that of the unmodified platinum electrode (0.45 mm^2^). The high active surface area of the electrode material was beneficial for better electrochemical conductivity and capacitance. The excellent electrochemical behavior of the modified electrode was attributed to the improved surface morphology and high surface area, which facilitated the electron diffusion at the electrode–electrolyte interface.

### 3.4. Electrochemical Response of Glutamate

**Figure 5:**
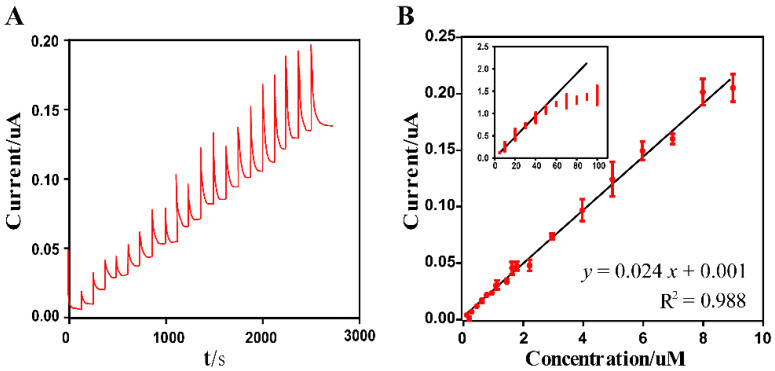
(**A**) Amperometric response curve of the modified electrode at 0.5 V upon successive injection of varying glutamate concentrations in a stirred phosphate buffer saline (PBS) buffer. (**B**) The calibration linear relationship between the oxidation peak currents and the glutamate concentrations (mean ± SD, *n* = 3).

Glutamate sensitivity and the linear range of the modified electrodes were characterized by chronoamperometry. The glutamate solution was gradually injected into the magnetic stirred PBS solution, gradually increasing the glutamate concentration from 50 nM to 150 μM. The amperometric responses in Figure 5A were recorded at a constant potential of 0.5 V. The linear relationship between the amperometric currents and glutamate concentration was depicted in Figure 5B. The current intensity changes caused by the glutamate concentration were fitted by the linear equation $I\;(\mu A)=0.024{\;C}_{Glu}\left(\mu M\right)\;+0.001$ (R^2^ = 0.988), when the concentration of glutamate was lower than 40 μM. The response currents reached the plateau stage as the glutamate concentration further increased, indicating that the glutamate concentration on the electrode surface had been saturated. The detection limit (LOD) was evaluated to be 41.33 nM for a signal-to-noise ratio of 3. The typical current intensity–time response (Figure 5A) and calibration linear plot (Figure 5B) indicated that the modified electrode exhibited a linear response to the glutamate concentration from 50 nM to 40 μM, corresponding to the glutamate concentration in the physiological conditions of the brain. 

The above results confirm that the surface modification of the nanomaterials led to an enormously improved electrocatalytic activity of the Pt wire electrode towards glutamate. The low LOD of the modified electrode allowed for efficient glutamate detection in an extracellular space, where the baseline glutamate concentrations are >2 μM [24]. Moreover, it also meant that the modified electrode had a better resolution ability for minor changes in glutamate concentration. 

### 3.5. Specificity, Reproducibility, and Stability

**Figure 6:**
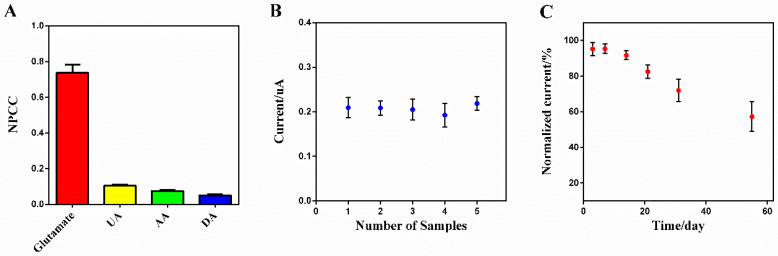
(**A**) Normalized peak current change of different analytes (glutamate, uric acid (UA), ascorbic acid (AA), and dopamine (DA)); (**B**) reproducibility for five samples; (**C**) storage stability for the modified electrode. Current responses in (**A**–**C**) are all recorded for analyte concentrations of 10 μM at 0.5 V (mean ± SD, *n* = 3).

In the real physiological environment, some co-existing electroactive substances may affect the response of electrodes, such as ascorbic acid (AA), dopamine (DA), and uric acid (UA). The anti-interference advantages were demonstrated in Figure 6A, which compared NPCC responses acquired as per the detection method of glutamate for three relevant electroactive species. Glutamate presented, by far, the highest electrochemical response for analyte concentration of 10 μM in artificial cerebrospinal fluid at 37 °C. These results indicated that the electrochemical response of glutamate could be quantified by the modified electrode in the presence of potentially interfering species under physiological conditions in the brain. This was probably ascribed to the Prussian blue layer that exhibited high sensitivity toward hydrogen peroxide as well as the chitosan and Nafion layer that block interferents by electrostatic repulsion [50].

In the stability and reproducibility tests, five electrodes were prepared using the same procedures and they were checked with chronoamperometry by determining 10 μM glutamate solutions in parallel. The electrodes were placed in deionized water and stored at 4 °C when not in use. The response current of five electrodes was recorded at different time intervals, and the residual activity was calculated relative to the initial signal. An acceptable relative standard deviation (RSD) value of 4.44% was observed (Figure 6B). 

Moreover, the operational stability of the electrodes was investigated by repetitive experiments. No significant changes in the response currents were found in the responses for up to 30 consecutive assays, which meant the electrochemical performance of the modified electrodes was stable, and the leakage and denaturation of the modified nanomaterials could be neglected. The electrodes also showed a prolonged storage stability, retaining 92.14% of the initial activity after 14 days and 57.31% after 50 days (Figure 6C). The excellent stability could be ascribed to the biocompatibility of chitosan, which could stabilize enzymes through surface entrapment and electrostatic interactions [52]. The strong attachment of nanoparticles to the electrode surface also empowers the cyclic life of the modified electrodes [53]. The above results indicate the suitability of the modified electrode for practical applications.

The applied potential, linear range and detection limit, storage stability, and reproducibility shown by the modified electrode were compared in Table 1 with the parameters reported in the literature [24,44,50,54,55,56]. It could be observed that the present electrode showed a high comprehensive performance. Working at a low applied potential was conducive for reducing interference and biological accumulation, while the small size of the modified electrode allowed for minimal damage with the implantation in the brain. In addition, the modified electrode exhibited a superior LOD at such a low detection potential and small size. Because of their miniature size, excellent stability, and low limit of detection, the modified electrodes provide potential application prospects for the in vivo detection of glutamate. In previous work, our team designed a novel Pt wire electrode based on rGO and AuNPs composites, and applied it on the detection of DA in the brain of rats [41]. By combining the glutamate microelectrode proposed in this study with the previous dopamine microelectrode, we could provide a new inexpensive multiplexed electrochemical detection system for simultaneously monitoring both glutamate and dopamine in future studies.

## 4. Conclusions

In summary, a highly sensitive glutamate electrode was designed and fabricated by the simple electrodeposition of reduced graphene oxide, Prussian blue nanocubes, gold nanoparticles, and chitosan onto Pt wires. The combination of rGO and metal nanoparticles contributed to the electron transfer and increased the effective electroactive surface areas as expected. Chitosan is conducive to maintaining the activity of glutamate oxidase and fixing the enzyme to the electrode more firmly. The modified electrode presents ascendant electrocatalytic properties toward glutamate with a detection limit of 41.33 nM and linearity over a physiologically useful concentration range. It also exhibits a high reproducibility (RSD = 4.44%) and retains up to 90% of its initial sensitivity for at least 14 days, as well as showing no observable decrease in sensor sensitivity after 30 continuous operations. Low applied potential and low dimension decrease bio-fouling and interference of electroactive compounds. All of the above results suggest that the proposed modified electrodes are easily fabricated and can find many potential applications in future neuroscience research.

## Figures and Tables

**Table 1:** Characteristics of various electrochemical microbiosensors reported for glutamate determination.

Biosensor Configuration	Electrode Type	E_ap_ (V) (Ag/AgCl)	Size	LOD (µM)	Linearity (µM)	Storage Stability	Reproducibility	References
PB; PoPD/ PEI/GluOx	Carbon fiber	0.05 V	d: 10 µm, l: 250 µm	<2.00	0–150	30 days: 90%	4.20%	[24]
CeO_2_/TiO_2_/AsOx/BSA/GluOx/Chit	Pt wire	0.60 V	d: 125 µm, l: 2 mm	0.49	0–50	10 days: 80%	<5.00%	[54]
Crbxl-RGO/PtNPs/Gldh/CHIT	Au plate	0.57 V (DPV)	(2 × 3.75) mm^2^	0.10	4–900	7 days: 91%	5.86%	[50]
PtNPs/NAEs	--	0.65 V	--	14.00	0–800	14 days: 98%	6.65%	[55]
Gel layer	Pt wire	0.60 V	d: 50 µm, l: 0.5 mm	0.05	0.5–100	150 days: 95%	--	[56]
cMWCNT/AuNPs/CHIT/GluOx	Au	0.20 V	0.36 cm^2^	1.60	5–500	7 days: 97.8%	--	[44]
rGO/PBNCs/AuNPs/CHIT/GluOx	Pt wire	0.50 V	d: 100 µm l: 6 mm	0.04	0.05–40	15 days: 92.14%	4.44%	This work

Abbreviations: LOD: limit of detection; E_ap_: applied potential; d: diameter; l: length; PoPD: poly-o-phenylenediamine; PEI: polyethyleneimine; CeO_2_: ceria oxide nanoparticles; TiO_2_: titania oxide nanoparticles; AsOx: ascorbate oxidase; BSA: bovine serum albumin; Crbxl-RGO: carboxyl terminated reduced graphene oxide; PtNPs: platinum nanoparticles; Gldh: glutamate dehydrogenase; NAEs: gold nanowire arrays; cMWCNT: carboxylated multiwalled carbon nanotubes.
